# *HuB* (*elavl2*) mRNA Is Restricted to the Germ Cells by Post-Transcriptional Mechanisms including Stabilisation of the Message by DAZL

**DOI:** 10.1371/journal.pone.0020773

**Published:** 2011-06-13

**Authors:** Sophie E. Wiszniak, B. Kate Dredge, Kirk B. Jensen

**Affiliations:** Discipline of Biochemistry, School of Molecular and Biomedical Science, University of Adelaide, Adelaide, Australia; Baylor College of Medicine, United States of America

## Abstract

The ability of germ cells to carry out a gene regulatory program distinct from the surrounding somatic tissue, and their capacity to specify an entire new organism has made them a focus of many studies that seek to understand how specific regulatory mechanisms, particularly post-transcriptional mechanisms, contribute to cell fate. In zebrafish, germ cells are specified through the inheritance of cytoplasmic determinants, termed the germ plasm, which contains a number of maternal mRNAs and proteins. Investigation of several of these messages has revealed that the restricted localisation of these mRNAs to the germ plasm and subsequent germ cells is due to cis-acting sequence elements present in their 3′UTRs. Here we show that a member of the Hu family of RNA-binding proteins, *HuB*, is maternally provided in the zebrafish embryo and exhibits germ cell specific expression during embryogenesis. Restriction of *HuB* mRNA to the germ cells is dependent on a number of sequence elements in its 3′UTR, which act to degrade the mRNA in the soma and stabilise it in the germ cells. In addition, we show that the germ cell specific RNA-binding protein DAZL is able to promote *HuB* mRNA stability and translation in germ cells, and further demonstrate that these activities require a 30 nucleotide element in the 3′UTR. Our study suggests that DAZL specifically binds the *HuB* 3′UTR and protects the message from degradation and/or enhances HuB translation, leading to the germ cell specific expression of HuB protein.

## Introduction

Zebrafish, a vertebrate with extrauterine development of the embryo, is currently one of the best animal models for understanding the molecular basis of germ cell specification. Briefly, germ cell specification involves the sequestration of maternally provided cytoplasmic determinants (specific protein and mRNA components). These cytoplasmic determinants (also known as germ plasm) are localised to areas of the embryo that will become the germ cells through cleavage divisions, and are set aside from the somatic lineages very early in development (reviewed in [1]).

The germ plasm is localised along the cleavage furrows of the 4-cell stage embryo, and by the 1000-cell stage (∼3 hours post-fertilisation (hpf)), has condensed into four separate cells. By the 4000-cell stage (∼4 hpf), the presumptive germ cells begin to divide, resulting in four separate clusters of cells distributed in the blastoderm. The four germ cell clusters migrate dorsally and along the anterior-posterior axis to arrive at their final position in two bilateral clusters at the anterior end of the yolk extension by 24 hpf, at the location where the future gonads will form [1].

A number of mRNA components of the germ plasm have been identified: *nanos* [2], *vasa* [3], *dead end* (*dnd*) [4], *dazl* [5] and *TDRD7* [6] are all specifically localised to the cleavage furrows of the 4-cell stage embryo. These five mRNAs all become specifically retained in the germ cells, suggesting that cis-acting elements in these mRNAs may drive both their initial localisation to the cleavage furrows and the subsequent retention specifically in the mature germ cells. Indeed, when a GFP open reading frame is fused to the 3′UTR region of all five messages and subsequently injected into zebrafish embryos, the localisation of these mRNAs, and the GFP protein expression pattern, recapitulates that of the endogenous genes; the injected RNAs are localised to and are stabilised in the germ cells, while being rapidly degraded in the soma [2], [3], [6]–[8].

In zebrafish, the microRNA miR-430 is expressed at the onset of zygotic transcription and is responsible for the downregulation of several hundred target mRNAs, most of which are maternally deposited messages, through deadenylation and clearance of these mRNAs [9]. miR-430 has been shown to directly target and degrade the *nanos*, *TDRD7* and *dazl* messages in the zebrafish soma [6], [8]. However, in the germ cells, these mRNAs are protected from degradation through sequence-specific interactions with a number of germ cell specific RNA-binding proteins. *Nanos* mRNA is protected by the germ cell specific RNA-binding protein DND [10], and *dazl* mRNA is protected by the RNA-binding protein DAZL [8], whereas *TDRD7* is able to be protected from degradation by both DND and DAZL [8], [10]. DND protects *nanos* and *TDRD7* mRNAs from degradation in the germ cells by binding to U-rich sequence elements in each 3′UTR and inhibiting the activity of miR-430 [10].

The mechanisms leading to the restricted localisation and presumptive expression of other germ cell specific mRNAs is not as well understood. For example, *vasa* [3], [6] and *dnd* [7] do not contain canonical miR-430 binding sites, suggesting that these mRNAs may be localised to the germ cells through unique post-transcriptional mechanisms.

The Hu protein family is a group of vertebrate sequence-specific RNA-binding proteins closely related to the ELAV and RBP9 proteins in *Drosophila* [11]. *Elav* is expressed specifically in the neurons and is required for the correct development and maintenance of the nervous system [12]. *Rbp9* is expressed in the nervous system [13], but is also expressed in the ovaries, and is required for correct oocyte differentiation [14]. Four *Hu* genes have been identified in vertebrates termed *HuA* to *HuD* (also known as *elav-like (elavl) 1–4* respectively). *HuA* (also known as *HuR* in humans [15]) is ubiquitously expressed, whereas the expression of *HuB*, *HuC* and *HuD* is restricted to developing and mature neurons [16]. *HuB* expression has also been shown in the testis in mouse [17], and in the testis, ovary and early embryo in *Xenopus* [18]. While four *Hu* genes have been identified in zebrafish, these include *HuA* and *HuG* (a likely ancestral gene duplication of *HuA*), both of which show ubiquitous expression, and *HuC* and *HuD*, both of which exhibit expression specifically in neurons [19]. No zebrafish *HuB* orthologue has been reported.

Since *HuB* has been identified and is conserved in species from *Xenopus* to mice and humans, it is highly likely that an unreported *HuB* gene exists in zebrafish. In this study, we identify the zebrafish orthologue of *HuB*. The zebrafish *HuB* mRNA is maternally provided, and is restricted to the germ cells by 24 hours of development. Given the critical role post-transcriptional regulatory mechanisms play in restricting the expression of a range of messages to the germ cells, we reasoned that this type of regulation might also play a role in the restricted expression of the *HuB* message. We report that the 3′UTR of *HuB* contains a number of cis-acting sequence elements that regulate the differential stability of *HuB* mRNA in the germ line and in the somatic tissues of zebrafish embryos. Using a scanning mutagenesis approach, we have identified a number of distinct sequence elements in the *HuB* 3′UTR that mediate destabilisation of the *HuB* mRNA in the soma. This approach also identified a separate 30 nucleotide sequence motif that is necessary for stabilisation of *HuB* mRNA specifically in the germ cells. Through a candidate screening approach, we have determined that the germ cell specific RNA-binding protein DAZL is able to promote increased stability and translation of the *HuB* mRNA in a manner that is dependent both on the ability of DAZL to bind RNA and on the presence of the 30 nucleotide stabilisation motif identified in the *HuB* 3′UTR. We propose a model for the germ cell specific expression of HuB protein in which *HuB* mRNA is targeted for degradation in the somatic tissue, yet is protected and translated in the germ cells through a specific interaction between DAZL and the 30 nucleotide *HuB* 3′UTR stabilisation motif.

## Results

### Zebrafish *HuB* mRNA is maternally provided and is expressed in the germ cells

**Figure 1:**
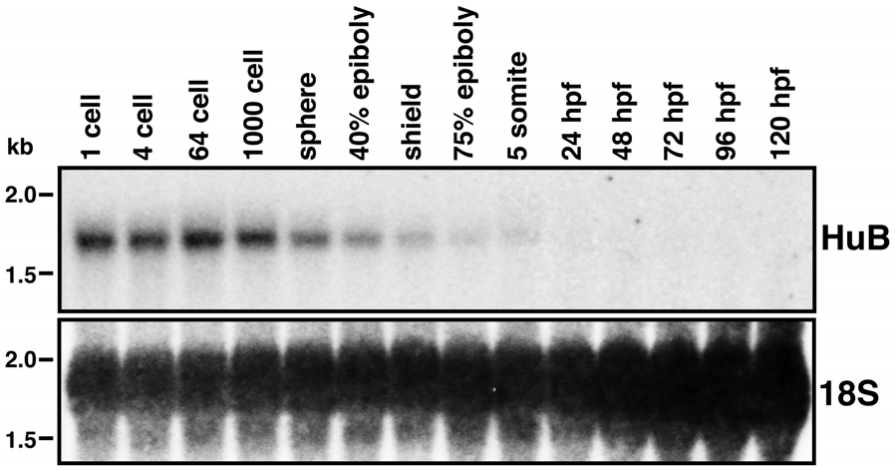
*HuB* mRNA expression over a time course of zebrafish embryonic development. Northern blot of a time course of zebrafish developmental stages, from 1-cell to 120 hours post fertilisation (hpf) as labelled. At each developmental stage, the total RNA from 30 embryos was used. Top panel: *HuB* mRNA expression levels. Bottom panel: 18S ribosomal RNA expression, serving as a loading control.

In order to identify the zebrafish orthologue of *HuB*, A TBLASTN search of the zebrafish genome was performed using the mouse HuB protein sequence, and a region of chromosome 22 was found to have high sequence homology. A zebrafish cDNA sequence (BC072716) identical to this region has been identified, as well as many spliced ESTs, suggesting this gene is likely to be zebrafish *HuB*. The RefSeq accession number for this gene is zgc:91918. To determine if *HuB* mRNA is expressed in the zebrafish embryo, Northern blot analysis was performed over a time course of developmental stages from the 1-cell stage to 120 hpf (Fig. 1). *HuB* mRNA is steadily expressed from the 1-cell stage to the 1000-cell stage with expression then rapidly decreasing and barely detectable by somitogenesis. This expression profile is similar to other known maternally expressed genes [2], [3], suggesting that *HuB* is a maternally deposited mRNA. Whole-mount *in situ* hybridisation data has been presented for zgc:91918 by Thisse et. al. in the ZFIN direct data submission database [20], and this demonstrates that *HuB* expression is partially restricted to the germ cells by 24 hpf.

### Post-transcriptional regulation of the *HuB* 3′UTR restricts HuB expression to the germ cells

**Figure 2:**
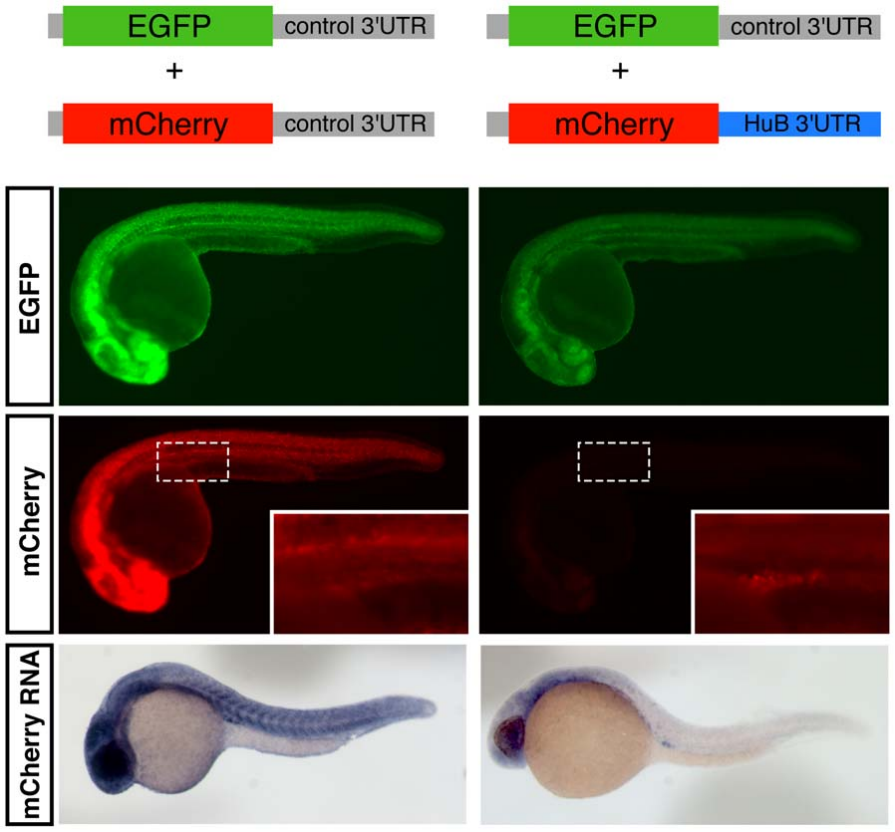
Post-transcriptional regulation of the *HuB* 3′UTR restricts HuB expression to the germ cells. Synthetic reporter RNAs, indicated schematically at top of figure, were transcribed *in vitro* and 200 pg of both the EGFP and mCherry reporter RNAs were microinjected into 1-cell stage embryos, which were then imaged at approximately 24 hpf. All EGFP images were taken at 2000 ms exposure time, and all mCherry images were taken at 150 ms exposure time. Top panels: EGFP protein expression; Middle panels: mCherry protein expression, insets show a higher magnification image of the germ cells (the dashed box indicates the position of the higher magnification image); Bottom panel: whole-mount *in situ* hybridisation using an antisense probe directed against the mCherry coding sequence.

To determine whether the *HuB* 3′UTR contains any cis-elements which may contribute to post-transcriptional regulation of the *HuB* mRNA, we designed a reporter system to measure mCherry protein expression from *in vitro* transcribed reporter RNAs containing the *HuB* 3′UTR sequence. These were compared to reporter RNAs containing the *Xenopus beta globin* 3′UTR as a control. The RNAs were injected into 1-cell stage zebrafish embryos along with an EGFP reporter RNA to serve as an injection volume control. At 24 hpf, the *HuB* 3′UTR reporter, in contrast to the control, showed significantly lower expression of mCherry protein in the somatic tissue (Fig. 2). The *HuB* 3′UTR reporter RNA levels were also significantly reduced compared to the control reporter RNA as determined by *in situ* hybridisation (Fig. 2). This suggests the *HuB* 3′UTR contains sequence elements which cause destabilisation of the RNA in the somatic tissue. In the germ cells, however, mCherry protein expression and reporter RNA stability were maintained, implying that the *HuB* 3′UTR must also contain sequence elements that promote stability and/or translational activation of the *HuB* mRNA specifically in the germ cells. This mechanism of directing stability of maternally deposited mRNAs specifically in the germ cells via sequence elements in the 3′UTR is also conserved in a number of other germ cell specific mRNAs in zebrafish, including *nanos* [2], *vasa* [3], *TDRD7* [6], *dnd* [7] and *dazl* [8].

### Cis-regulatory elements control differential *HuB* mRNA stability in the soma and germ cells

**Figure 3:**
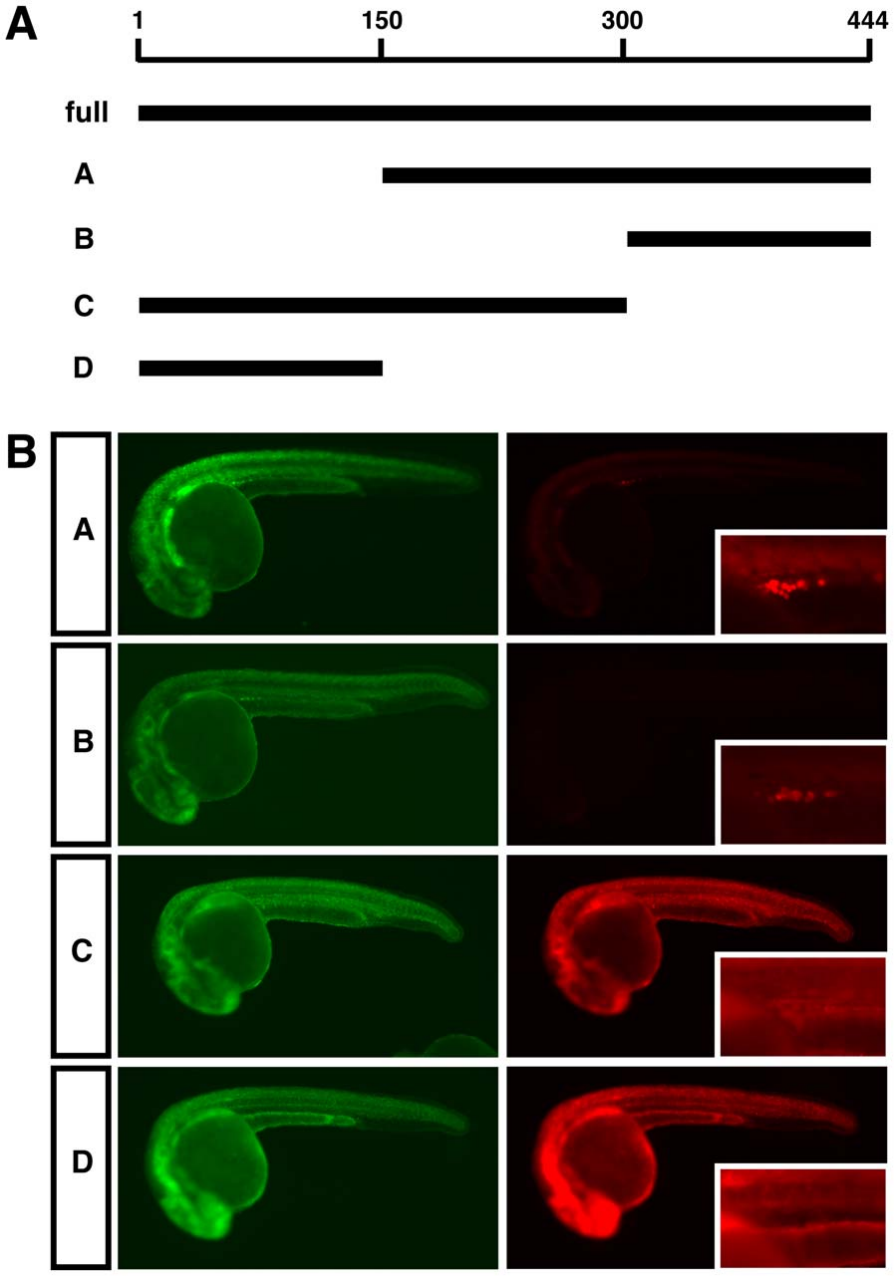
The last 144 nt of the *HuB* 3′UTR are necessary and sufficient for post-transcriptional regulation of *HuB* mRNA. (A) Schematic representation of the four *HuB* 3′UTR deletion reporter RNAs constructed, termed A, B, C and D. The 3′UTR regions present in each of the reporters are indicated by black bars, and are shown relative to the full-length 3′UTR of 444 nucleotides at the top. (B) Expression of EGFP and mCherry protein in embryos injected with the indicated deletion mutant reporters. 200 pg of each reporter was injected. All EGFP images were taken at 2000 ms, and all mCherry images were taken at 150 ms. Higher magnification images of mCherry expression in the germ cells are shown as insets.

In order to determine what sequence elements in the *HuB* 3′UTR are necessary for the observed somatic instability and germ cell specific stabilisation, we created four deletion constructs of the *HuB* 3′UTR, termed A, B, C and D, as indicated in Fig. 3A. Compared to the full length *HuB* 3′UTR reporter, mutants C and D showed increased expression of mCherry in the somatic tissue (Fig. 3B). This implies that these mutant RNAs are stable in the somatic tissue, and therefore the destabilising elements must be located within the last 144 nucleotides of the 3′UTR. Mutants A and B, both of which contain these last 144 nucleotides, recapitulated the behaviour of the full length *HuB* 3′UTR, showing both low levels of mCherry expression in the somatic tissue (implying somatic instability of the RNAs), and enriched expression of mCherry protein in the germ cells (Fig. 3B). Since the B mutant was the shortest sequence able to replicate the function of the full length 3′UTR, this reporter RNA was chosen for further study. We have confirmed that the cells specifically expressing the B reporter are germ cells, as co-injection of the B RNA with an EGFP-*nanos* 3′UTR reporter shows co-expression of both reporters in the same cells (Fig. S1). The *nanos* 3′UTR has previously been shown to direct germ cell specific expression at 24 hpf [2].

**Figure 4:**
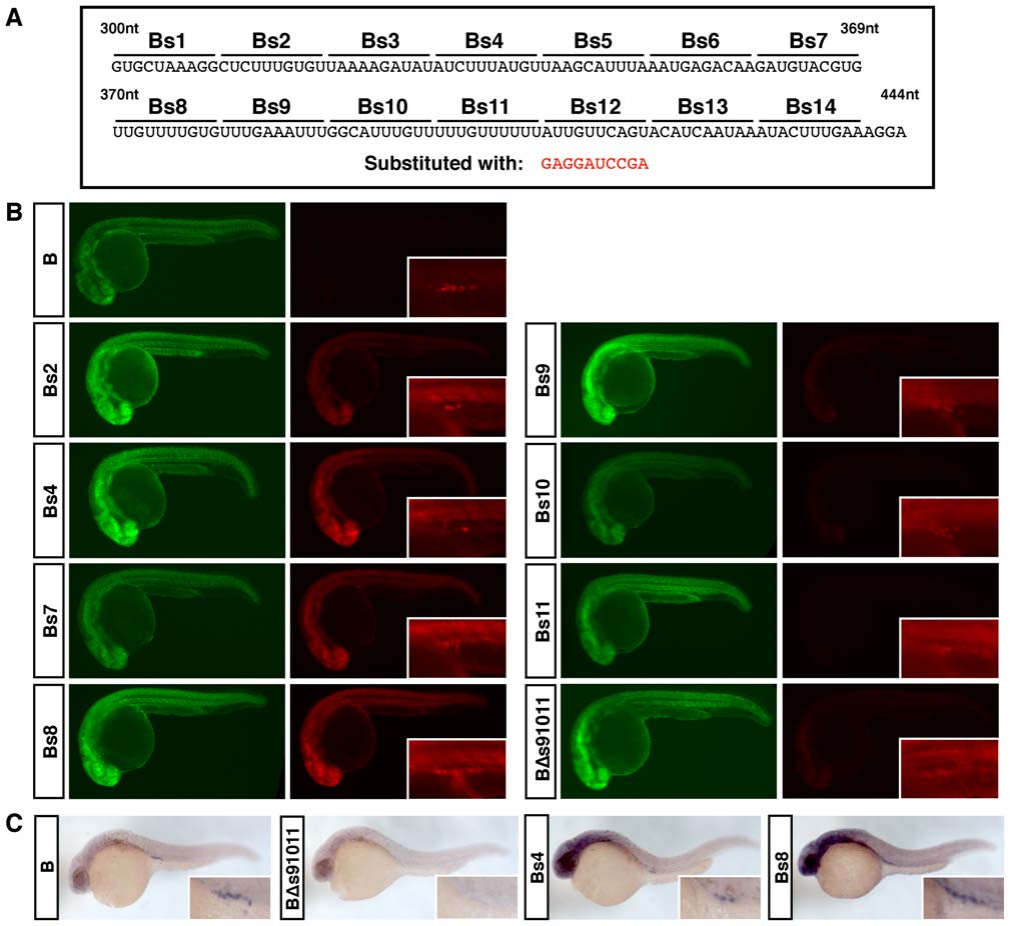
Cis-regulatory elements control differential *HuB* mRNA stability in the soma and germ cells. (A) 3′UTR sequence of the B deletion reporter. Base substitutions were made in 10 nucleotide blocks to create the B substitution mutants Bs1–Bs14. Each 10 nucleotide block was substituted with the sequence GAGGATCCGA. (B) Expression of EGFP and mCherry protein in embryos injected with the indicated Bs substitution mutant reporters. 200 pg of each reporter was injected. All EGFP images were taken at 2000 ms, and all mCherry images were taken at 150 ms. Higher magnification images of mCherry expression in the germ cells are shown as insets. (C) Whole-mount *in situ* hybridisation using an antisense probe directed against the mCherry coding sequence. Higher magnification images of germ cells are shown as insets.

To further identify the sequence elements within the B region that are important for the post-transcriptional regulation of the *HuB* 3′UTR, a substitution mutagenesis approach was taken. Sequential 10-nucleotide blocks of the B region were substituted with the sequence GAGGAUCCGA. In total, 14 mutants were made, termed Bs1 to Bs14 as indicated (Fig. 4A). The Bs substitution mutant RNAs were each injected into 1-cell stage embryos and compared to the activity of the wild type B reporter. Several substitution mutations caused an increase in mCherry expression (Fig. 4B) and RNA stability (Fig. 4C) in the somatic tissue, implying that these substitutions must be disrupting sequence elements which are normally important for somatic instability of the *HuB* 3′UTR. Most notably, these were Bs2, Bs4, Bs7 and Bs8 (Fig. 4B). Bs2 and Bs4 share an almost identical sequence motif (UCUUUGUGU and UCUUUAUGU respectively) which possibly represent multiple binding sites for the same microRNA or RNA-binding protein. Bs7 and Bs8 do not share this exact sequence motif, but Bs8 is clearly U-rich like Bs2 and Bs4, and may also share a common binding site. Alternatively, given that Bs7 and Bs8 are directly adjacent to each other, they may possibly encompass a single binding site for a destabilising factor that may or may not be the same as that interacting with Bs2 and Bs4. MicroRNA miR-430 is known to promote the deadenylation and clearance of other germ cell specific mRNAs, such as *nanos*, *TDRD7*, and *dazl*, in the somatic tissue of zebrafish [6], [8], however a canonical miR-430 binding site is not present in the *HuB* 3′UTR. No known zebrafish microRNAs [21], [22] were found to have seed sequence homology to any of the destabilising elements identified, and therefore the mechanism of *HuB* somatic instability remains unclear.

Several substitutions were found to cause a decrease in the expression of mCherry in the germ cells, yet leave the absence of somatic expression of the reporter protein unaffected. These were Bs9, Bs10 and Bs11 (Fig. 4B). Since these mutations were adjacent to each other, we hypothesised that the entire 30 nucleotides may act as a single binding site for a factor that maintains the stability and/or promotes translational activity of *HuB* mRNA in the germ cells. Therefore, a mutant termed BΔs91011 was designed to replace the entire 30 nucleotides with a single GAGGAUCCGA sequence. This mutation almost completely abolished mCherry expression in the germ cells (Fig. 4B), and *in situ* hybridisation confirmed that this was due to a lack of reporter RNA in the germ cells (Fig. 4C). This suggests the 30 nucleotide U-rich 91011 region is essential for germ cell specific stabilisation of *HuB* mRNA. The RNA-binding proteins DND, DAZL and HuB have been shown to relieve microRNA-mediated repression of *nanos*, *TDRD7* and *dnd* mRNAs respectively in the germ cells [8], [10], [23]. We therefore hypothesised that the 91011 region identified is a binding site for a germ cell specific factor, such as an RNA-binding protein, which binds and protects the *HuB* mRNA from destabilisation.

The remaining Bs reporters were also injected into one cell stage embryos, but did not show any significant differences in their somatic and germ cell specific expression when compared to the parental B RNA (Fig. S2).

### DAZL is able to stabilise *HuB* mRNA

**Figure 5:**
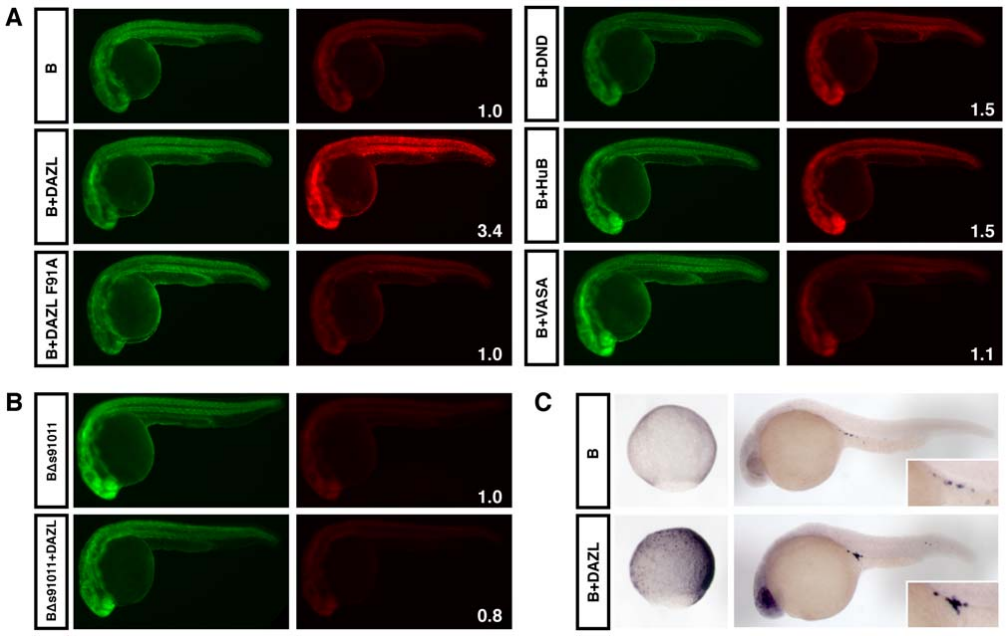
DAZL protein is able to stabilise the *HuB* mRNA. (A) Expression of EGFP and mCherry protein in embryos injected with 150 pg each of EGFP and mCherry-B reporter RNAs, either alone (labelled B), or with ubiquitous overexpression of HA-tagged DAZL, DND, HuB (400 amol RNA) or VASA (200 amol RNA) as labelled. All EGFP images were taken at 2000 ms and all mCherry images were taken at 500 ms. The fold change in the somatic expression of mCherry from the B reporter-protein combination compared to the B reporter alone is indicated as an inset number in white. (B) mCherry-BΔs91011 reporter RNA +/− overexpression of DAZL. (C) Whole-mount *in situ* hybridisation using an antisense probe directed against the mCherry coding sequence. Embryos at 9 hpf are shown in the left panels (sagittal view with dorsal side to the right), and embryos at 24 hpf are shown in the right panels with germ cells shown as insets.

In order to identify the factor responsible for stabilising *HuB* mRNA specifically in the germ cells, a set of candidate germ cell specific RNA-binding proteins were overexpressed in the somatic tissues of the zebrafish embryo to see whether this misexpression of a germ cell specific RNA-binding protein would lead to a subsequent increase in the somatic expression of the mCherry-B reporter RNA. The western blot of all HA-tagged overexpressed proteins confirmed that all were expressed and were of the expected size (Fig. S3). Fig. 5A shows that overexpression of DND, HuB and VASA caused a modest 1.1 to 1.5 fold increase in mCherry expression in the somatic tissue when compared to the B reporter alone. However, overexpression of DAZL caused a marked increase in somatic expression of mCherry, with a 3.4 fold increase of mCherry protein compared to the B reporter alone. A DAZL RNA-binding mutant containing a phenylalanine to alanine substitution at amino acid position 91 (DAZL F91A) [24], was tested in the same experiment and found to be unable to cause any increase in mCherry expression (Fig. 5A). DAZL overexpression was also tested in the context of the BΔs91011 reporter, and was found to be unable to lead to an increase in mCherry expression (Fig. 5B). Whole-mount *in situ* hybridisation demonstrates an increase in mCherry-B reporter RNA levels in the somatic tissue of 9 hpf embryos upon DAZL overexpression, which strongly suggests that DAZL acts to stabilise the mCherry-B reporter RNA (Fig. 5C). This increase in reporter RNA levels was less apparent at 24 hpf, however there was still a marked increase in reporter RNA levels in the germ cells when DAZL was overexpressed.

Taken together, these experiments suggest that DAZL is able to stabilise and possibly activate translation of the mCherry-B reporter RNA when expressed in the same tissue, and that this stabilisation is dependent on both the RNA-binding ability of DAZL and on the presence of the 91011 sequence element within the 3′UTR. Furthermore, these findings are consistent with the hypothesis that DAZL is the protein factor responsible for binding to the *HuB* 3′UTR, causing stabilisation of this mRNA in the germ cells.

While our experiments do not conclusively show that the interaction between DAZL protein and the *HuB* 3′UTR is direct, the inability of the F91A DAZL mutant to increase mCherry levels and the dependence of this increase upon the B reporter having an intact 91011 sequence argues that DAZL does indeed bind directly to the B reporter RNA. Further proof of this RNA-protein interaction would require in vitro experiments with purified DAZL protein and RNA substrates.

## Discussion

In this study, we have identified the zebrafish *HuB* gene, shown that *HuB* mRNA is maternally provided in the embryo, and that the message is highly abundant from the 1-cell stage to early somitogenesis stages. *HuB* expression has also been shown in the early embryo in *Xenopus* [18], suggesting that *HuB* has an important and evolutionary conserved role in early embryogenesis, independent of the more characterised role of *HuB* in neurogenesis.

Whole-mount *in situ* hybridisation has also demonstrated that *HuB* mRNA expression is restricted to the germ cells later in development. Other germ cell specific mRNAs such as *nanos* [2] and *vasa* [3] show extremely similar expression profiles to *HuB*, and the restriction of a number of these maternally deposited mRNAs to the germ cells has been found to be mediated by cis-acting sequence elements present in the 3′UTR of these messages [2], [3].

A mCherry RNA reporter under the regulatory control of the *HuB* 3′UTR, when injected into 1-cell stage zebrafish embryos, was highly enriched specifically in the germ cells, as was the localisation of the injected reporter RNA. This suggests that the *HuB* mRNA is endogenously post-transcriptionally regulated, and this regulation is controlled by the *HuB* 3′UTR. The enrichment of the reporter RNA in the germ cells and absence in the soma, as determined by *in situ* hybridisation, also suggests that the RNA is degraded in the somatic tissue, yet specifically stabilised in the germ cells. Furthermore, a deletion screen identified the last 144 nucleotides of the 3′UTR (a construct termed ‘B’) as being both necessary and sufficient to recapitulate the post-transcriptional regulatory effect of the full-length 3′UTR. Interestingly, we find that the ‘B’ region of the zebrafish *HuB* 3′UTR is well conserved in human, mouse, chicken and *Xenopus*, implying that this sequence likely plays an important post-transcriptional role in all vertebrate species.

The ‘B’ region of the *HuB* 3′UTR was further dissected by creating a series of substitution mutants, which identified two classes of *cis*-acting RNA elements. The first class caused an increase in mCherry reporter protein expression and reporter RNA stabilisation in the somatic tissue. RNA destabilisation may be mediated by a number of mechanisms, including microRNA-mediated gene silencing, or perhaps binding of an RNA-binding protein that promotes RNA degradation. While the microRNA miR-430 is responsible for promoting the deadenylation and subsequent degradation of several germ cell specific mRNAs in the somatic tissue, a canonical miR-430 binding site is not present in the *HuB* 3′UTR [6], [9]. In fact, no known zebrafish microRNAs [21], [22] were found to have seed sequence homology to any of the destabilising elements identified in the substitution mutatagenesis analysis. However, it is likely that the destabilisation of *HuB* mRNA in the somatic tissue is at least in part dependent, either directly or indirectly, on microRNA function as a GFP-*HuB* 3′UTR reporter RNA (zgc:91918) showed an increase in somatic GFP expression in embryos deficient in both maternal and zygotic Dicer activity [9]. A recent report has characterised four non-canonical miR-430 sites in the HuB 3′UTR, three of which lie at the 5′ end of the 3′UTR (and thus reside in our D RNA reporter), while the fourth coincides with the sequence present in the Bs5 mutant [25]. While it is possible that our A RNA deletion reporter, which would lack the three 5′ non-canonical miR-430 sites, is more stable than the full-length *HuB* 3′UTR, it is clear from our results that the most important elements controlling differential stability in the soma and in the germ cells reside in the last 144 nt of the *HuB* 3′UTR. However, Mickoleit *et al* did not explicitly test the regions in the *HuB* 3′UTR that we have identified as affecting somatic mRNA stability as potential microRNA targets; thus we believe the mechanism regulating the somatic instability of the *HuB* mRNA is unresolved.

The second class of mutations identified caused a decrease in mCherry reporter protein expression in the germ cells, but did not affect the regulation of the reporter RNA in the somatic tissue. Because these mutations, Bs9, Bs10 and Bs11, lie directly adjacent to each other, the entire 30 nucleotide region (91011) may act as a single binding site for a factor which is responsible for maintaining the stability and/or promoting translational activity of *HuB* mRNA in the germ cells. To identify if any germ cell specific proteins can act to stabilise *HuB* mRNA in the germ cells, candidate germ cell specific RNA-binding proteins were overexpressed in the somatic tissue to see whether this would lead to a subsequent increase in the somatic expression of the mCherry-B 3′UTR reporter RNA. Overexpression of DAZL caused a marked increase in expression of mCherry protein in the somatic tissue, and also dramatically increased the stability of the reporter RNA as assayed by *in situ* hybridisation at 9 hpf. This suggests that DAZL is a likely candidate for stabilising *HuB* mRNA specifically in the germ cells. The function of DAZL in increasing the stability and translation of the mCherry-B 3′UTR reporter RNA was also dependent on the ability of DAZL to bind RNA, and on the presence of the 91011 sequence element in the *HuB* 3′UTR. Several independent studies have investigated the RNA-binding specificity of the DAZL protein. Studies using the zebrafish DAZL protein have identified a consensus binding sequence of ‘GUUC’ [24], whereas studies using mouse DAZL protein have identified a more loosely defined binding element of stretches of U interspersed with G or C residues [26]–[28]. Although the 91011 element in the *HuB* 3′UTR does not contain an exact ‘GUUC’ sequence, it does contain several repeats of the motif GUUU, and so fits the criteria of being a high-affinity DAZL binding site. While our results do not conclusively demonstrate that the interaction between DAZL protein and the *HuB* 3′UTR is direct, the requirement of an intact 91011 sequence within the B reporter to achieve a DAZL-mediated increase in reporter mCherry levels, and the inability of the F91A DAZL mutant to stimulate any increase in reporter protein synthesis argues that DAZL does indeed bind directly to the B reporter RNA. Further proof of this RNA-protein interaction would require in vitro experiments with purified DAZL protein and RNA substrates.

DAZL has been shown to stimulate translation of reporter mRNAs in *Xenopus* oocytes via a mechanism that is dependent on the ability of DAZL to recruit poly(A) binding protein (PABP) [29]. DAZL is also able to increase poly(A) tail length of *TDRD7* mRNA in zebrafish embryos, another germ cell specific mRNA, thus promoting increased mRNA stability and/or translational activation [8]. It is possible then that DAZL may function by interacting with target mRNAs to promote stability and stimulate translation by combinatorially inducing polyadenylation and recruiting PABPs.

It is also possible that DAZL may function cooperatively with other germ cell specific RNA-binding proteins such as DND and HuB, as these were shown to modestly increase translation of the *HuB* mRNA. Interestingly, in *Xenopus* the HuB orthologue ElrB has been shown to synergise with XDE (the *Xenopus* orthologue of DND) in the stabilisation of *XDE* mRNA [23], so perhaps overexpression of DAZL with DND or HuB may cause an even more dramatic increase in expression of the reporter RNA.

The elucidation of the mechanisms of germ cell specific expression of the *HuB* mRNA is an important finding, for it reveals mechanisms of post-transcriptional regulation that are distinct from that of the two best understood germ cell specific mRNAs, *nanos* and *TDRD7*, in which DND and DAZL can relieve miR-430-mediated repression of these messages in germ cells [8], [10]. Indeed, the germ cell specific *vasa* and *dnd* messages are not targeted by miR-430 [3], [6], [7]; nothing is yet known about how these two mRNAs are degraded in the somatic tissue. Therefore the identification of specific destabilising elements in the *HuB* 3′UTR may lead to the discovery of microRNA or protein factors that may also be responsible for destabilising one or more of these messages in the soma. Knockdown of *HuB* in zebrafish embryos using morpholino oligonucleotides does not affect germ cell migration or survival [25], and thus elucidation of HuB protein function in zebrafish germ cell biology will require further study.

In summary, the identification of *HuB* as a germ cell specific mRNA, and the determination of the post-transcriptional mechanisms responsible for this specific expression is an important first step in understanding how HuB and other germ cell RNA-binding proteins contribute to germ cell development and function. An important future direction will be to identify RNA targets of these germ cell specific RNA-binding proteins to gain further insight into the molecular mechanisms of germ cell specification and development.

## Materials and Methods

### Fish husbandry

For all experiments, male and female zebrafish of the Tübingen, AB or pet shop sourced wild type strains were used. Embryos were collected by natural spawning and staged according to [30]. All work followed the Australian Code of Practice for the Care and Use of Animals for Scientific Purposes, and was carried out as described in ethics Project # S-050-2006.

### Constructs used

All plasmids contain a T7 promoter and *Xenopus beta-globin* 5′UTR sequence, a multiple cloning site used for the insertion of ORFs (described below), a A_(30)_ poly(A) sequence, and a unique restriction site 3′ of the poly(A) sequence for linearisation of plasmids before *in vitro* transcription. *EGFP-control 3′UTR* is derived from pXT7 (a gift of the laboratory of Judith Eisen, Univ. of Oregon) and contains the EGFP coding sequence followed by *Xenopus beta-globin* 3′UTR. The following plasmids were derived from the pSP64 Poly(A) (Promega) vector backbone in which the SP6 promoter was replaced with the T7 promoter and *Xenopus beta-globin* 5′UTR sequence from pXT7: *mCherry-control 3′UTR* contains N-terminal FLAG tag, mCherry coding sequence followed by *Xenopus beta-globin* 3′UTR; *mCherry-HuB 3′UTR and derivatives* contains an N-terminal FLAG tag and mCherry coding sequence followed by *HuB* 3′UTR or deletions/substitutions as indicated in the text. Deletions and nucleotide substitutions were generated by PCR. Bs 10-nucleotide substitutions contained a BamHI restriction site. The plasmids for DAZL (NM_131524.1), DAZLF91A, DND (NM_212795.1), HuB (NM_001002172.1), and VASA (NM_131057.1) all contain an N-terminal HA tag, a full-length coding sequence, and the *Xenopus beta-globin* 3′UTR, except DND, which has a C-terminal HA tag. mCherry plasmid [31] was obtained from the laboratory of Roger Y. Tsien, UCSD. Probes for the HuB and 18S Northern were synthesised by random priming. The template for HuB was a dsDNA fragment corresponding to the 3′UTR, while the 18S probe was generated with PCR primers 5′-ATCGCTCCATCCGTTACTTG-3′ and 5′-GCTTATGACCTGCGCTTACTG-3′.

### Transcription and microinjection of reporter mRNAs

Plasmids for *in vitro* transcription were first linearised 3′ of the encoded poly(A) region and then transcribed using the mMESSAGE mMACHINE T7 Ultra kit (Ambion) following the manufacturers recommendations. All RNAs were microinjected into 1-cell stage embryos in approximate volumes of 3 nl.

### Whole-mount *in situ* hybridisation

Antisense mCherry probe was synthesised in an *in vitro* transcription reaction using linearised pCDNA3-mCherry plasmid (containing the entire mCherry coding sequence) as template. *In situ* hybridisation was performed as described by Thisse, C and Thisse, B [32]. Images within figures were always probed together and developed for the same amount of time.

### Quantitation of changes in fluorescent protein abundance

ImageJ [33] software was used to measure fluorescence intensities. Mean pixel intensity was determined for a rectangle above the yolk extension, and background values from a dark region next to the embryo were subtracted. EGFP and mCherry values were measured from a minimum of 10 embryos per experiment, and the mCherry value for each embryo was divided by the EGFP value to normalise to the injection control. The average of the normalised mCherry/EGFP ratios with overexpressed germ cell protein was divided by the average of the normalised mCherry/EGFP ratios for B alone to give the fold change. The fold change was always calculated with normalised mCherry/EGFP values from within the same experiment.

## Supporting Information

Figure S1 **The B 3′UTR reporter is expressed in germ cells.** Synthetic reporter RNAs were transcribed *in vitro* and both the EGFP-nanos and mCherry-B reporter RNAs were microinjected into 1-cell stage embryos, which were then imaged at approximately 24 hpf. Top panels: EGFP and mCherry protein expression; insets show a higher magnification image of the germ cells; Bottom panel: image overlays of both EGFP and mCherry protein expression showing coincident expression; image intensity of the mCherry expression was increased to allow for visualisation of protein co-expression in yellow.
(TIF)

Figure S2 **Bs substitution reporters that show no significant differences relative to the parental B reporter.** Expression of EGFP and mCherry protein in embryos injected with the indicated Bs substitution mutant reporters. 200 pg of each reporter was injected. All EGFP images were taken at 2000 ms, and all mCherry images were taken at 150 ms. Higher magnification images of mCherry expression in the germ cells are shown as insets.
(TIF)

Figure S3 **Overexpression of HA-tagged germ cell proteins.** Western blot of overexpressed HA-tagged proteins. Total protein was extracted from 8 embryos at 6 hpf and probed with anti-HA antibody 6E2 (Cell Signaling Technology).
(TIF)
