# Supplementary figures and images for: HuB (elavl2) mRNA Is Restricted to the Germ Cells by Post-Transcriptional Mechanisms including Stabilisation of the Message by DAZL
Source: PLoS One. 2011 Jun 13;6(6):e20773. doi: 10.1371/journal.pone.0020773 (PMC3113899; doi:10.1371/journal.pone.0020773)

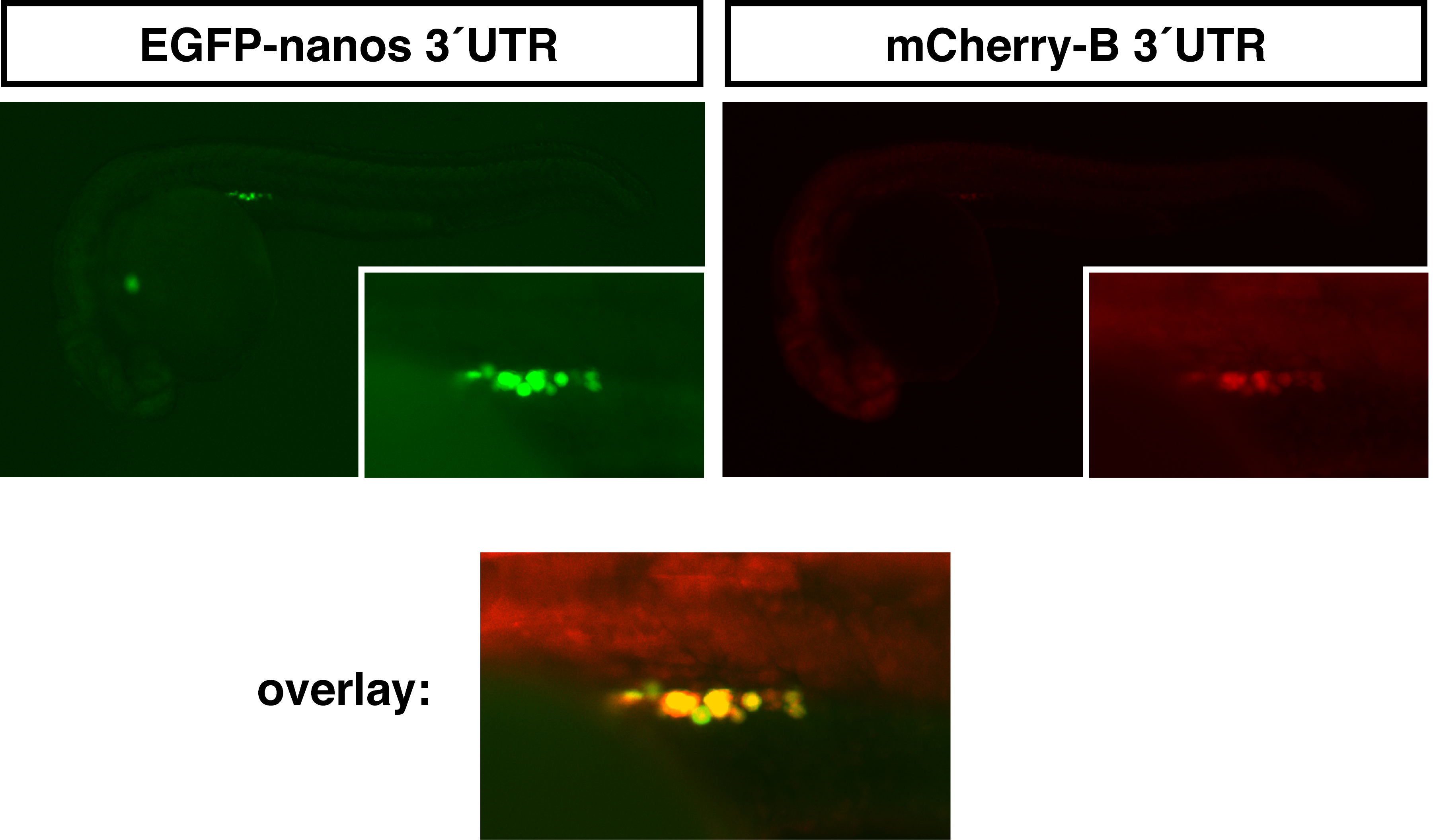

Supplement: Figure S1 — The B 3′UTR reporter is expressed in germ cells. Synthetic reporter RNAs were transcribed in vitro and both the EGFP-nanos and mCherry-B reporter RNAs were microinjected into 1-cell stage embryos, which were then imaged at approximately 24 hpf. Top panels: EGFP and mCherry protein expression; insets show a higher magnification image of the germ cells; Bottom panel: image overlays of both EGFP and mCherry protein expression showing coincident expression; image intensity of the mCherry expression was increased to allow for visualisation of protein co-expression in yellow. (TIF) [file pone.0020773.s001.tif]

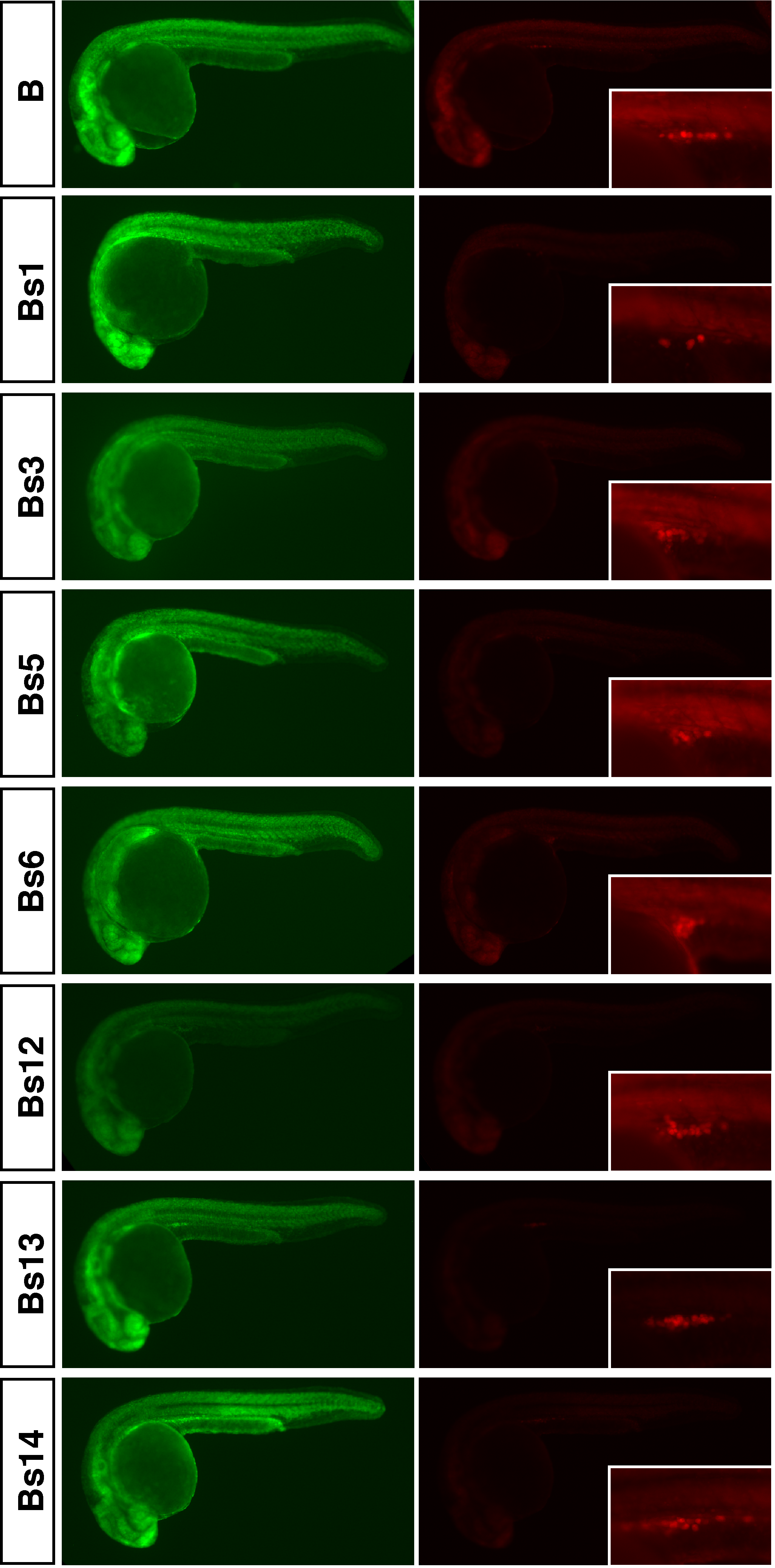

Supplement: Figure S2 — Bs substitution reporters that show no significant differences relative to the parental B reporter. Expression of EGFP and mCherry protein in embryos injected with the indicated Bs substitution mutant reporters. 200 pg of each reporter was injected. All EGFP images were taken at 2000 ms, and all mCherry images were taken at 150 ms. Higher magnification images of mCherry expression in the germ cells are shown as insets. (TIF) [file pone.0020773.s002.tif]

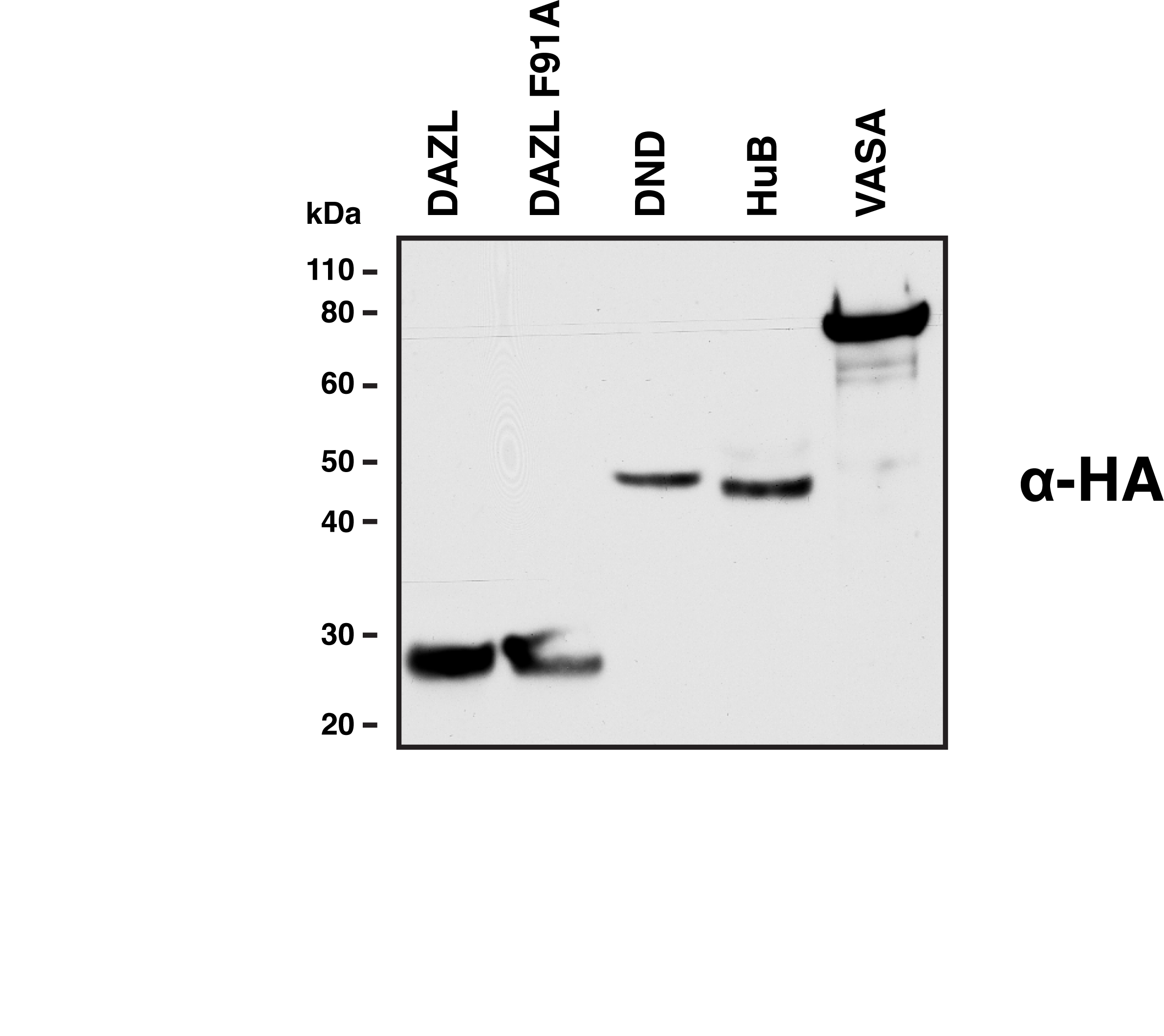

Supplement: Figure S3 — Overexpression of HA-tagged germ cell proteins. Western blot of overexpressed HA-tagged proteins. Total protein was extracted from 8 embryos at 6 hpf and probed with anti-HA antibody 6E2 (Cell Signaling Technology). (TIF) [file pone.0020773.s003.tif]
